# Mortality among MDR-TB Cases: Comparison with Drug-Susceptible Tuberculosis and Associated Factors

**DOI:** 10.1371/journal.pone.0119332

**Published:** 2015-03-19

**Authors:** Kocfa Chung-Delgado, Sonia Guillen-Bravo, Alejandro Revilla-Montag, Antonio Bernabe-Ortiz

**Affiliations:** Escuela de Medicina, Universidad Peruana de Ciencias Aplicadas, Lima, Peru; Public Health Agency of Barcelona, SPAIN

## Abstract

**Background:**

An increase in multidrug-resistant tuberculosis (MDR-TB) cases is evident worldwide. Its management implies a complex treatment, high costs, more toxic anti-tuberculosis drug use, longer treatment time and increased treatment failure and mortality. The aims of this study were to compare mortality between MDR and drug-susceptible cases of tuberculosis, and to determine risk factors associated with mortality among MDR-TB cases.

**Methods and Results:**

A retrospective cohort study was performed using data from clinical records of the National Strategy for Prevention and Control of Tuberculosis in Lima, Peru. In the first objective, MDR-TB, compared to drug-susceptible cases, was the main exposure variable and time to death, censored at 180 days, the outcome of interest. For the second objective, different variables obtained from clinical records were assessed as potential risk factors for death among MDR-TB cases. Cox regression analysis was used to determine hazard ratios (HR) and 95% confidence intervals (95%CI). A total of 1,232 patients were analyzed: mean age 30.9 ±14.0 years, 60.0% were males. 61 patients (5.0%) died during treatment, whereas the MDR-TB prevalence was 19.2%. MDR-TB increased the risk of death during treatment (HR = 7.5; IC95%: 4.1–13.4) when compared to presumed drug-susceptible cases after controlling for potential confounders. Education level (p = 0.01), previous TB episodes (p<0.001), diabetes history (p<0.001) and HIV infection (p = 0.04) were factors associated with mortality among MDR-TB cases.

**Conclusions:**

MDR-TB is associated with an increased risk of death during treatment. Lower education, greater number of previous TB episodes, diabetes history, and HIV infection were independently associated with mortality among MDR-TB cases. New strategies for appropriate MDR-TB detection and management should be implemented, including drug sensitivity tests, diabetes and HIV screening, as well as guarantee for a complete adherence to therapy.

## Introduction

Tuberculosis (TB) was declared a “global emergency” by the World Health Organization (WHO). Recent surveillance data shows a more frequent report of multidrug-resistant (MDR-TB) and extensively drug-resistant tuberculosis (XDR-TB) cases. The WHO estimated 450,000 new cases of MDR-TB in 2012 [1]. About 6% of these drug-resistant cases were XDR-TB [2,3]. This growth trend has notably been observed in Peru: currently, 5.3% of all diagnosed tuberculosis cases are MDR-TB [4], yet studies suggest that this value might be underestimated [5–7].

The management and treatment of MDR-TB is complex; it includes an elevated treatment cost [8], the use of highly toxic anti-tuberculosis drugs with potential adverse effects [9], longer treatment time [6], and it burdens an increased treatment failure and mortality rate [10,11]. In Peru, MDR-TB mortality varies from 20% to 55% [12,13], much greater compared to the 4.5% to 17% mortality of sensitive-TB [14,15].

Some factors have been previously described as associated with high mortality in tuberculosis cases such as drug-resistant forms [11], HIV infection [16–18], diagnosis of diabetes mellitus [19], malignancy [20], daily alcohol intake and malnutrition [21]. The risk of death during treatment is directly correlated with the number of risk factors present [22]. However, further investigation is needed assessing independent factor association with MDR-TB mortality, as its prevalence and burden worldwide becomes more evident—especially in high-incidence epidemiologic scenarios. This information is vital in order to propose and implement new, appropriate strategies for the control of this “global emergency” [1]. Thus, this investigation aims to: (a) compare mortality rates between multidrug-resistant and drug-susceptible cases of tuberculosis, and (b) determine independent risk factors associated with mortality during treatment among MDR-TB cases.

## Methods

### Study design

A retrospective cohort study was conducted at hospitals and TB control health centers chosen by convenience of the National Strategy for Prevention and Control of Tuberculosis (ESNPCT) in Lima, Peru. The data collected were all the records of patients enrolled in the ESNPCT and their follow-up information during their anti-tuberculosis treatment until therapy completion or death (censorship).

### Study settings and patients

We retrieved information from all patients aged 18 years and above that began and completed their anti-tuberculosis treatment for a non-XDR pulmonary TB disease in the San Juan de Miraflores—Villa María del Triunfo Health Network (SJM-VMT) in Lima, Peru between January 2000 and December 2012. If the anti-tuberculosis treatment initiation date was not available in the clinical record, the patient was excluded from analysis. In addition, treatment failures were also excluded due to the risk of misclassification in the exposure of interest.

### Variable definition

The main outcome of interest for both objectives was time to death. Death during follow-up was defined as the deceased condition of a patient during treatment confirmed by the presence of a death notification form or by explicit indication in the clinical record. For the survival analysis, the follow-up time was censored at 180 days. Therefore, the time until event was defined as the time elapsed, in days, from the beginning of the anti-tuberculosis treatment to the date of completion or death. If the patient was transferred or abandoned therapy, then time until event was censored at the moment of transfer of therapy abandonment.

For the first objective, type of tuberculosis was dichotomized as MDR-TB or drug-susceptible TB according to information of clinical records, based especially upon clinical assessment and the use of first- or second-line drugs, at the beginning of treatment. Thus, in the case of MDR-TB forms, diagnosis and classification was made following international standards and national guideline protocols (culture and drug susceptibility tests). This information was confirmed by checking treatment schemes as well as laboratory results. The remaining patients, those without a culture or drug-susceptibility test, were categorized as presumed drug-susceptible TB cases.

For both objectives, several other variables were included: sex (male vs. female), age (in tertiles), education level (≤6 years vs. 7–10 years vs. ≥11 years), previous TB episodes (0 vs. 1 vs. 2 or more), diabetes history (yes vs. no), anemia (positive vs. negative), HIV infection (positive vs. negative), self-reported smoking (yes vs. no), self-reported alcohol use (yes vs. no), self-reported illicit drug use (yes vs. no) and body mass index (BMI) (underweight vs. normal vs. overweight/obesity). All variables were assessed at the beginning of the anti-tuberculosis treatment.

### Procedures and data collection

Sputum smear is indicated to those in whom tuberculosis is suspected, according to Peruvian National Guidelines [23]. The ESNPCT enrolls any patient with a positive result and the patient starts first-line anti-tuberculosis treatment. Culture and drug-susceptibility tests are performed in order to investigate a possible MDR-TB case if any of the following risk factors are present in a patient: confirmed MDR-TB contact, immunodeficiency (HIV or diabetes history), treatment relapse within six months after an active TB disease, TB cases treated multiple times, health workers, convicts, illegal drug users, contact with a person who died with TB or failed to complete treatment, hospitalization, irregular treatment schemes and adverse drug reaction to the treatment [23]. The patient’s treatment scheme is then adapted accordingly, if necessary. Patients without these risk factors are given first-line drugs. They are considered drug-susceptible TB cases for this analysis. Patients are followed and monitored during the duration of their whole treatment.

Data was collected directly from the patient’s clinical onto a single database. Only the investigators performed data collection during this study.

### Sample size and statistical analysis

Power and Sample Size (PASS) software was used for sample size calculation. Assuming a confidence level of 95% and a power of 80%, a minimum total of 1090 patients were required to find a Hazard Ratio (HR) of 2.0 or over for any variable of interest. Calculations assumed a MDR-TB prevalence of 5.4% and an expected mortality of 6%.

After a double data entry process, statistical analysis was performed using STATA for Windows (STATA Corp, College Station, TX, USA). Patients who failed treatment during follow-up were excluded from analysis. Mortality curves were obtained through the Kaplan-Meier method. Each variable was evaluated with a Log-Rank test to verify potential association with mortality. Cox Regression models (crude and adjusted) were used to compare MDR-TB vs. drug-susceptible TB mortality, reporting Hazard Ratio (HR) with a 95% confidence interval (95%CI). Factors associated with mortality among MDR-TB cases were also assessed using Cox regression methods, assessing proportional hazard assumptions.

### Ethics

This investigation was approved by the Ethics Committee of the Universidad Peruana de Ciencias Aplicadas (UPC), Lima, Peru and by the Institutional Ethics and Investigation Committee of Hospital María Auxiliadora, head of the SJM-VMT Health Network. The identity of participants was not recorded in order to guarantee anonymity.

## Results

Data from a total of 1,242 patients was recorded. Of them, 10 (0.8%) were excluded due to treatment failure. Therefore, a total of 1,232 records were included in the analysis. The mean age was 30.9 ± 14.0 years, and the proportion of males was 60.0%. Of the enrolled patients, 236 (19.2%) were reported as MDR-TB cases and 61 (5.0%) died during time of follow-up. Of the total deceased, 44 (72.1%) corresponded to MDR-TB cases.

A detailed description of the population characteristics according to the outcome of interest is shown in Table 1. Factors associated with overall TB mortality were: age, education level, previous TB episodes, diabetes history, HIV infection, self-reported drug use, BMI and type of tuberculosis.

**Table 1:** Population characteristics according to the outcome of interest.

	Alive	Deceased	p-value*
	(n = 1,171)	(n = 61)	
**Sex, n (%)**			
Female	472 (95.7%)	21 (4.3%)	0.29
Male	699 (94.6%)	40 (5.4%)	
**Age [tertiles], n (%)**			
Low tertile	451 (98.5%)	7 (1.5%)	<0.001
Middle tertile	351 (94.9%)	19 (5.1%)	
High tertile	369 (91.3%)	35 (8.7%)	
**Education level, n (%)**			
≥11 years	607 (97.6%)	15 (2.4%)	<0.001
7–10 years	364 (94.6%)	21 (5.4%)	
≤6 years	200 (88.9%)	25 (11.1%)	
**Previous TB episodes, n (%)**			
No previous episodes	929 (98.6%)	13 (1.4%)	<0.001
1 episode	202 (90.6%)	21 (9.4%)	
2+ episodes	40 (59.7%)	27 (40.3%)	
**Diabetes history, n (%)**			
No	1,143 (96.0%)	48 (4.0%)	<0.001
Yes	28 (68.3%)	13 (31.7%)	
**Anemia, n (%)**			
No	1,018 (95.5%)	48 (4.5%)	0.05
Yes	153 (92.2%)	13 (7.8%)	
**HIV infection, n (%)**			
No	1,157 (95.5%)	55 (4.5%)	<0.001
Yes	14 (70.0%)	6 (30.0%)	
**Smoking, n (%)**			
No	1,008 (95.6%)	47 (4.4%)	0.05
Yes	163 (92.1%)	14 (7.9%)	
**Alcohol use, n (%)**			
No	946 (95.5%)	45 (4.5%)	0.13
Yes	224 (93.3%)	16 (6.7%)	
**Drug use, n (%)**			
No	1,074 (95.6%)	49 (4.4%)	<0.001
Yes	96 (88.9%)	12 (11.1%)	
**Body mass index, n (%)**			
Normal	798 (96.3%)	31 (3.7%)	0.002
Overweight/obese	139 (95.2%)	7 (4.8%)	
Underweight	233 (91.0%)	23 (9.0%)	
**Type of tuberculosis, n (%)**			
Drug-susceptible	979 (98.3%)	17 (1.7%)	<0.001
MDR	192 (81.4%)	44 (18.6%)	

* P-values were calculated using Log-rank test.

**Fig 1:**
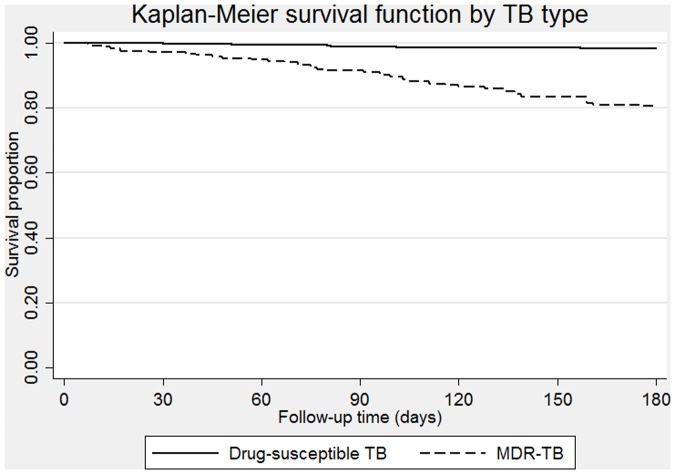
MDR-TB survival curves using Kaplan-Meier estimates.

MDR-TB was associated with an increased mortality in the crude model (p < 0.001), as detailed in Table 2. Results were consistent after adjusting for age, sex, and education level, as well as all other potential confounders (diabetes history, anemia, HIV infection, self-reported smoking, self-reported alcohol use, self-reported drug use, and BMI). The final result after adjusting for these variables was HR = 7.5 (95%CI: 4.1–13.4). The survival function based on type of tuberculosis can be seen in Fig. 1.

**Table 2:** Cox regression model for mortality in tuberculosis patients.

Type of Tuberculosis	Crude model	Adjusted model 1*	Adjusted model 2**
	HR (95%CI)	HR (95%CI)	HR (95%CI)
Drug-susceptible TB	1 (Reference)	1 (Reference)	1 (Reference)
DR-TB	11.7 (6.7–20.5)	9.5 (5.4–16.8)	7.5 (4.1–13.4)

* Adjusted for age, sex and education level

** Adjusted for age, sex, education level, anemia, diabetes history, HIV infection, smoking, alcohol use, drug use, and BMI.

Factors independently associated with mortality within the group of MDR-TB cases were: education level (p = 0.02), previous TB episodes (p < 0.001), diabetes history (p < 0.001) and HIV infection (p = 0.04). Detailed information is shown in Table 3.

**Table 3:** Factors independently associated with mortality among MDR-TB patients.

	Bivariable model	Multivariable model*
	HR (95%CI)	HR (95%CI)
**Sex**		
Female	1 (Reference)	
Male	1.05 (0.57–1.94)	
**Age [tertiles]**		
Low tertile	1 (Reference)	
Middle tertile	2.23 (0.80–6.18)	
High tertile	**3.78 (1.45–9.88)**	
**Education level**		
11+ years	1 (Reference)	1 (Reference)
7–10 years	1.40 (0.62–3.18)	2.13 (0.89–5.07)
6 or less years	**3.40 (1.67–6.92)**	**3.06 (1.43–6.55)**
**Previous TB episodes**		
No previous episodes	1 (Reference)	1 (Reference)
1 episode	2.77 (0.90–8.51)	**3.13 (1.01–9.73)**
2+ episodes	**10.23 (3.58–29.27)**	**7.79 (2.59–23.47)**
**Diabetes history**		
No	1 (Reference)	1 (Reference)
Yes	**4.10 (2.15–7.85)**	**5.42 (2.66–11.04)**
**Anemia**		
No	1 (Reference)	
Yes	0.44 (0.16–1.22)	
**HIV infection**		
No	1 (Reference)	1 (Reference)
Yes	2.33 (0.83–6.51)	**3.18 (1.05–9.69)**
**Smoking**		
No	1 (Reference)	
Yes	0.40 (0.12–1.29)	
**Alcohol use**		
No	1 (Reference)	
Yes	0.51 (0.21–1.20)	
**Body mass index**		
Normal	1 (Reference)	
Overweight/obese	1.14 (0.46–2.84)	
Underweight	1.84 (0.97–3.50)	

Significant estimates (p<0.05) are in bold

* This model only includes variables independently associated with mortality.

## Discussion

The main conclusions that arise from this investigation are: (a) MDR-TB is associated with an increased risk of mortality during treatment when compared to presumed drug-susceptible cases even after adjusting for several potential confounders, and (b) lower education level, having previous TB episodes, previous history of diabetes, and HIV infection were factors independently associated with a higher mortality during treatment among MDR-TB cases.

### MDR-TB and mortality

Our results demonstrate that multidrug-resistant strains of tuberculosis substantially increase the risk of death when compared to presumed drug-susceptible TB cases during anti-tuberculosis treatment. Recent studies have found similar findings where MDR-TB is associated with a higher mortality rate [24,25], with HR estimates ranging from 7.8 to 8.5. Our results have been controlled for several confounders, which makes a stronger adjusted association.

The cause behind this increased risk of death attributable to multidrug-resistant forms is multifactorial. MDR-TB is commonly seen as a co-morbidity to other conditions such as HIV infection, diabetes and chronic kidney disease, as well as particular lifestyles including alcohol abuse, smoking, drugs use, etc [21]. Furthermore, MDR-TB has been associated with elevated rates of treatment failure and relapse, which directly increases the proportions of death amongst these patients [10]. The longer exposure to anti-tuberculosis drugs might also increase the risk of death as antibiotics used for MDR-TB have proven higher toxicity profiles and greater incidence of adverse effects [10]. There is no doubt, then, that a multidisciplinary management of drug-resistant TB is needed and of great importance.

Scientific literature indicates that drug-resistant tuberculosis has increased over time. In 1990, Peru was believed to have a MDR-TB prevalence of 1%; however, recent publications have reported a prevalence of up to 5.3% [5], and our study suggests approximately 20% might have MDR-TB. This rise in resistant tuberculosis proportion over time might be related to many factors, namely treatment abandonment and other epidemiologic circumstances such as the existence of clusters or overcrowded households—especially in resource-limited settings [26]; nonetheless, the use of new diagnostic tools for detecting drug-resistant strains may be exposing the actual context of MDR-TB, reassuring the existence of this public health problem.

### Factors independently associated with mortality among MDR-TB cases

*Education level*: According to our results, MDR-TB patients with lower education level have a greater risk of death during specific treatment. Of importance, education has been associated with a better adherence to treatment as it increases awareness regarding the disease [26]. Education has also been recognized as an economic status marker; in this case, lower education may be associated with lack of resources, overcrowding and unsanitary conditions.

*Previous TB episodes*: A greater number of previous TB episodes has been similarly associated with a higher risk of death. Patients under multiple regimens of anti-tuberculosis treatment might create greater antibiotic resistance with the subsequent development of MDR- and XDR-TB cases [2]. This causal link between number of past episodes and the appearance of drug-resistant strains has been thoroughly mentioned in scientific literature [6, 12]. However, the reasons for the association reported in this study require further investigation.

*Diabetes history*: Diabetes history increases the risk of mortality because it reduces cellular immunity, which favors the progression of the disease [28]. This association has been demonstrated in previous studies [19, 20]. Given the high frequency of diabetes as a co-morbidity among MDR-TB patients [27], screening for this disease should be encouraged in order to guarantee an appropriate treatment and control of both pathologies, as its awareness can reach up to 60% [28].

*HIV Infection*: HIV infection was also associated with a greater death proportion in MDR-TB patients. Investigations regarding this topic state that around 12% to 20% of all tuberculosis-related deaths may be attributed to an HIV co-infection [22,29], even if highly active antiretroviral therapy (HAART) has shown to reduce the overall mortality [18,30]. The results in our analysis might be the reflection of the HIV screening protocol done to all MDR-TB cases that was recently implemented by the ESNPCT.

### Strengths and limitations

This study includes over 200 cases of MDR-TB under a directly-observed treatment scheme (DOTS), which is a notable strength. In addition, sample size allowed us to control for several variables in our proposed models. Nevertheless, this study has several limitations. First, non-TB related deaths such as accidents or other chronic diseases could have been included as the death certificate stating specific cause of death was not available. Although this might propose a bias in our findings, the results are greatly compatible and comparable with literature. Second, it was not possible to obtain drug-susceptibility test data for non-MDR TB participants. Drug-susceptibility and cultures are performed only in those with high risk or suspected MDR-TB, yet are not done in patients without such risk factors. Third, the starting point of evaluation, especially in the situation of MDR-TB cases, can be an issue as potentially resistant forms of tuberculosis may need longer periods of diagnosis and have previously administered treatment. Therefore, our results might be overestimated. Nonetheless, our findings are comparable to the investigations previously mentioned—many of which consider the beginning of treatment as their starting point of analysis. In addition, data from the ESNPCT maintains a certain level of objectivity and uniformity as it bases its clinical records on clinical forms and notifications. Fourth, there is a possibility of misclassification of MDR-TB into the drug-susceptible group as MDR-TB is widely under-diagnosed. Nevertheless, our findings show a high proportion of MDR-TB cases (about 20%), greater than previous studies using cultures to detect resistant cases [11] and even greater than the official ESNPCT surveillance report [5]. Finally, the data concerning smoking, alcohol and drug use was collected as a self-report notification. No clinical parameters, questionnaires or scales were applied to properly define the use of these substances. Due to its small amount, we believe that the risk of misclassification is non-differential, thus allowing the report of a true association.

## Conclusions

In this study, MDR-TB is associated with increased risk of death during treatment when compared to presumed drug-susceptible TB cases. Lower education, number of previous TB episodes, diabetes history and HIV infection were independently associated with mortality in MDR tuberculosis.

MDR-TB patients should have a more consistent follow-up: patients should be thoroughly educated on their condition, making special mention in guidelines regarding the treatment adherence, possible adverse effects and infectivity of the disease; a thorough clinical history should be made to investigate possible cases of past TB episodes; diabetes and HIV screening and control should be incorporated into the follow-up evaluation. As such, the already-existing DOTS program should be modified and renewed to fit the requirements of MDR-TB patients and not vice-versa, with particular interest in countries where the MDR- and XDR-TB incidence rates are in ascent.
